# Supplementary material for: The genome sequence of the brimstone moth, Opisthograptis luteolata (Linnaeus, 1758)
Source: Wellcome Open Res. 2022 Sep 12;7:227. [Version 1] doi: 10.12688/wellcomeopenres.18101.1 (PMC9523282; doi:10.12688/wellcomeopenres.18101.1)
Supplement: Peer review history

# Peer review: Reviewer response for version 1

**DOI:** 10.21956/wellcomeopenres.20072.r52336

**Published:** 2022-09-29

**Authors:** Jun Li

**Affiliations:** 1 College of Life Sciences, Huaibei Normal University, Huaibei, China

In this article, the genomes of *Opisthograptis luteolata* (Linnaeus, 1758) were sequenced. The  nuclear genome is 363 megabases and mitochondrial genome is 16.7 kilobases in length. Most of the assembly sequence were assigned to 31 chromosomal-level scaffolds which were corresponding to 30 autosomes and one sex chromosome. These are helpful for us to understand the moth.

Are sufficient details of methods and materials provided to allow replication by others?

Yes

Is the rationale for creating the dataset(s) clearly described?

Yes

Are the datasets clearly presented in a useable and accessible format?

Yes

Are the protocols appropriate and is the work technically sound?

Yes

Reviewer Expertise:

Animal genetics

I confirm that I have read this submission and believe that I have an appropriate level of expertise to confirm that it is of an acceptable scientific standard.

# Peer review: Reviewer response for version 1

**DOI:** 10.21956/wellcomeopenres.20072.r52338

**Published:** 2022-09-27

**Authors:** Leidys Murillo-Ramos

**Affiliations:** 1 Department of Biology, Universidad de Sucre, Sucre, Colombia

The genome sequence of the brimstone moth, *Opisthograptis luteolata *(Linnaeus, 1758) 

 The authors present a genome assembly from Ophisthograptis luteolata (Geometridae), commonly known as the brimstone moth. The genome was sequenced from a fresh male sample collected near Chalet, Wytham, Berkshire, UK. The DNA extraction, library preparation and sequencing procedures followed the latest protocols in genome processing. The genome assembly was carried out with Hifiasm and following well known protocols. The genome sequencing resulted in 363 MB in 33 sequence scaffolds. As stated by the authors, the majority of the assembly (99.99%) is scaffolded into 31 chromosomal pseudomolecules with the Z sex chromosome assembled. The complete mitochondrial genome was also assembled and is 16.7 kilobases in length. Moreover, the data was compared to the lepidoptera_obd10 reference set from BUSCO dataset and they retrieved 98,3% of completeness. In figure 2, the authors present a great summary of the results.  The plot is divided into 1,000 size-ordered bins around the circumference with each bin representing 0.1% of the 363,201,500 bp assembly. I do not have any further comments, and I encourage the publication of this research. I personally congratulate the authors for the initiative and collaborative effort that will be available to researchers and public interested in moths.

Are sufficient details of methods and materials provided to allow replication by others?

Yes

Is the rationale for creating the dataset(s) clearly described?

Yes

Are the datasets clearly presented in a useable and accessible format?

Yes

Are the protocols appropriate and is the work technically sound?

Yes

Reviewer Expertise:

Molecular systematics

I confirm that I have read this submission and believe that I have an appropriate level of expertise to confirm that it is of an acceptable scientific standard.
